# Building improvement capability in frontline staff: a UK perspective

**DOI:** 10.1192/bji.2020.26

**Published:** 2020-11

**Authors:** Daniel Maughan, Gurpreet K. Reen, Jill Bailey

**Affiliations:** 1 Consultant Psychiatrist and Medical Lead, Oxford Healthcare Improvement Centre, Oxford Health NHS Foundation Trust, UK. Email: danielmaughan@nhs.net; 2 Researcher, Oxford Healthcare Improvement Centre, Oxford Health NHS Foundation Trust, UK.; 3 Acting Clinical Director, Oxford Healthcare Improvement Centre, Oxford Health NHS Foundation Trust, UK.

**Keywords:** Quality improvement, patient safety, clinical governance

## Abstract

This paper gives a narrative account of how the Oxford Healthcare Improvement Centre has embedded continuous quality improvement (CQI) across both mental health and community services in Oxford, UK. The aim of the centre is to develop capability across healthcare services, with frontline staff leading CQI independently. The paper discusses the various methods employed to achieve this aim, including the provision of training, mentoring and support to those undertaking improvement work, alongside developing the required governance for CQI.

Oxford Health NHS Foundation Trust is a mental health and community healthcare organisation targeting a population of around 2.5 million people in the South of England. The organisation provides specialist services to support patients of all ages with physical or mental healthcare needs. Services include acute psychiatric care, older adult psychiatric provision, eating disorder and forensic services, as well as a wide range of physical healthcare services provided in both a hospital and community setting. The trust is closely connected with primary care, community pharmacies and local authorities to deliver more cohesive care to patients. The organisation also collaborates with a number of research universities, including the University of Oxford and Oxford Brooks University.

## Oxford Healthcare Improvement Centre

Continuous quality improvement (CQI) uses a set of techniques originating in industrial settings that have been adopted in healthcare over the past 30 years with the intention of improving healthcare systems and processes and improving patient care.^1,2^ CQI can lead to the implementation of interventions ranging from checklists and team huddles, through to more complex ‘care bundles’ or changes in clinical pathways.^1^ The aim is to make changes that become embedded in routine practice and are eventually sustained overtime.^3^

Over the past 2 years at Oxford Health NHS Foundation Trust, we have been building the capability for CQI across a range of mental health and community settings (Box 1). We have established the Oxford Healthcare Improvement (OHI) Centre, which provides training, mentoring and support to those undertaking quality improvement work, as well as establishing governance structures to build improvement capability.

Box 1 Example of improvement projects across different healthcare settings in organisations supported by Oxford Healthcare Improvement

Psychiatric in-patient services

• Reducing the number of young people self-harming on an adolescent in-patient psychiatric unit
• Reducing the hours nursing staff spend constantly observing adult in-patients at risk of harm on a psychiatric intensive care unit
• Reducing restrictive practices for patients (e.g. restraint and seclusion) on three adult forensic psychiatric units, including an intellectual disability forensic service

Community settings

• Preventing falls among older adults across five community hospitals
• Reducing staff barriers to using end-of-life care plans with older adults
• Improving the process of mental health referrals for older adults

Corporate and organisation-wide projects

• Improving and evaluating the adult eating disorder in-patient pathway
• Reducing clinician time taken to complete mental health assessment forms on electronic health record systems
• Embedding a culture of psychological safety within all levels of the organisation

### Team

The OHI team lies across a range of disciplines, including clinicians, researchers and analysts, who facilitate the improvement work in the trust. This was an intentional design to ensure good engagement with clinical teams alongside rigour in the quality improvement approach and evaluation of improvement work, which are common reasons for failure if done poorly.^1,4^

### Approach to quality improvement

Carrying out CQI in healthcare is a complex activity.^4^ Leading CQI successfully requires many skills, including a working knowledge of the different methodologies of CQI, good social skills, as well as resilience and perseverance.^3^ It can therefore be challenging to lead successful improvement projects and harder still to embed capability within a healthcare organisation with frontline staff leading CQI independently.^4^

At OHI, we support teams to lead all elements of an improvement project: analysing the problem, choosing measures, designing and implementing sequential changes, evaluating the data and sustaining improvement. Considerable time is spent in the analytical stage to help the team understand the ‘system’ that they wish to improve, a step that was previously given only cursory attention or missed entirely. Different team members contribute their understanding of the problem through a range of lenses to ensure better identification of potential solutions. Teams are also encouraged to engage patients and families throughout the improvement process, and co-produce improvement work with patients when possible.^3^ OHI supports teams for approximately a year, but this can vary depending on the nature of the improvement work, internal and external pressures on the team, and the motivation of the staff. After this time, OHI offers to formally review the success and challenges of the project and provide continued support until the team feels capable of leading improvement work independently.

### Training

Training is another major component in building CQI capability within a system.^3^ OHI delivers two substantial training courses, one aimed at frontline staff (6-months duration) and the other at future leaders of improvement (1-year duration). Both courses require the participant to lead a piece of improvement work with their team while being closely supervised by OHI. Training is structured to match the different stages of the improvement work and incorporates research, leadership and team-working skills that are essential to successful CQI. The OHI team also offers bespoke training for teams that are interested in leading their own CQI projects, as well as providing an introduction to CQI in established training programmes within the organisation (Box 2).

Box 2 Training provided by Oxford Healthcare Improvement (OHI)

Dedicated continuous quality improvement (CQI) programmes

• 1-year advanced training course on CQI:
  • aimed at leaders in the organisation from clinical and non-clinical services
  • current size of cohort: 20
  • teaching and project time: 2 days per month
  • teaching by OHI team and external speakers
  • one-to-one and group supervision with OHI team
  • trainees required to lead an improvement project
  • OHI hosts a celebration day at the end of training to share projects with executives and the wider organisation
• 6-month foundation training course to introduce CQI:
  • aimed at frontline and non-clinical staff in the organisation
  • current size of cohort: 15
  • teaching and project time: 1.5 days per month
  • teaching by OHI team
  • one-to-one supervision with OHI team
  • trainees required to lead an improvement project
  • OHI hosts a celebration day at the end of training to share projects with executives and the wider organisation
• 2–5 days of bespoke training:
  • available on request to any team within the organisation interested in CQI
  • typical size of cohort: 15
  • teaching by 2–4 members of OHI team
  • trainees may carry out an independent improvement project or an improvement project coached by OHI

Integrated CQI training

• 2 days’ training within a 10-month leadership and CQI programme:
  • aimed at leaders in the organisation
  • teaching by 2–4 members of OHI team
  • trainees required to write a 4000-word Masters-level assignment on their improvement work
• 1 h session within a 6-month preceptorship programme:
  • aimed at newly qualified healthcare professionals developing an awareness of CQI
  • teaching by 2–4 members of OHI team

### Governance

A critical aspect of building capability has been to establish governance structures for CQI within the organisation, both to enable strategic alignment of improvement work and to actively manage and support CQI activities. These can be difficult to establish because requirements from regulatory bodies often focus clinical leaders on compliance with standards rather than on CQI. OHI has identified clear executive leadership within the organisation to develop CQI strategy. Progress in improvement work is reported to this leadership team at senior-level quality meetings, alongside lower-level meetings that can support CQI activities more directly. Sponsorship by senior staff provides visible support and troubleshooting for teams who are testing their improvement ideas and empowers frontline teams to lead improvement work and pursue CQI training offered by OHI.

### Example of an improvement project: self-harm reduction on an adolescent psychiatric ward

Approximately 2 years ago, OHI was approached by the frontline team of a 12-bed child and adolescent psychiatric service concerned about the self-harm happening on their ward. Research in other psychiatric units shows that self-harm has a negative physical and psychological impact not only on the young person harming, but also on adolescents observing the incident, who may mimic the behaviour or become distressed, and on staff using restrictive practices to de-escalate the situation.^5^ Similar issues were raised by the frontline team of this unit.

An OHI improvement lead met regularly with the team to coach them through their improvement journey. A considerable time was spent exploring the reason for self-harm incidents on the ward. This included speaking with staff and patients, as well as looking for patterns in routinely collected data. This highlighted that around 70% of self-harm incidents on the ward occurred in the evening. With a better understanding of the problem, the team decided to add a twilight nursing shift to support adolescents during this vulnerable time. OHI helped the team to seek senior support in order to implement this change. Only once the shifts became embedded into routine practice and data were available to test this change was the team encouraged to make further changes, including the development of a structured evening activities programme on the ward. The team have successfully reduced the number of adolescents self-harming on the ward since adopting the CQI methodology to make improvements. The team have shared their learning with other members of the organisation and their project is currently being written up for publication. The frontline team also feel capable of leading improvement projects independently and are looking to make improvements in other areas on the ward.

## Reflections

Despite initial challenges of embedding and promoting the OHI Centre within Oxford Health NHS Foundation Trust, the work of OHI is being recognised by both senior and frontline staff as critical to improving the quality of patient care. Improvement work within the organisation is led by teams that will be affected by the changes through coaching and mentoring by OHI staff. Teams are encouraged to begin CQI work with a thorough understanding of the problems in practice, ideally informed by research and data when available, and by working closely with patients throughout the improvement journey. Training in CQI also plays a key role in building capability at all levels of the organisation. Senior support to help troubleshoot and empower frontline teams is a critical part of success in CQI within the organisation. OHI aims to continue building capability for improvement work across the organisation and ensure that these changes are sustained over time.

## Supplementary material

For supplementary material accompanying this paper visit https://doi.org/10.1192/bji.2020.26. click here to view supplementary material
